# Study of Integrated Heterogeneous Data Reveals Prognostic Power of Gene Expression for Breast Cancer Survival

**DOI:** 10.1371/journal.pone.0117658

**Published:** 2015-02-27

**Authors:** Richard E. Neapolitan, Xia Jiang

**Affiliations:** 1 Department of Preventive Medicine, Northwestern University Feinberg School of Medicine, Chicago, Il 60611, United States of America; 2 Department of Biomedical Informatics, University of Pittsburgh, Pittsburgh, PA, 15213, United States of America; Ohio State University Medical Center, UNITED STATES

## Abstract

**Background:**

Studies show that thousands of genes are associated with prognosis of breast cancer. Towards utilizing available genetic data, efforts have been made to predict outcomes using gene expression data, and a number of commercial products have been developed. These products have the following shortcomings: 1) They use the Cox model for prediction. However, the RSF model has been shown to significantly outperform the Cox model. 2) Testing was not done to see if a complete set of clinical predictors could predict as well as the gene expression signatures.

**Methodology/Findings:**

We address these shortcomings. The METABRIC data set concerns 1981 breast cancer tumors. Features include 21 clinical features, expression levels for 16,384 genes, and survival. We compare the survival prediction performance of the Cox model and the RSF model using the clinical data and the gene expression data to their performance using only the clinical data. We obtain significantly better results when we used both clinical data and gene expression data for 5 year, 10 year, and 15 year survival prediction. When we replace the gene expression data by PAM50 subtype, our results are significant only for 5 year and 15 year prediction. We obtain significantly better results using the RSF model over the Cox model. Finally, our results indicate that gene expression data alone may predict long-term survival.

**Conclusions/Significance:**

Our results indicate that we can obtain improved survival prediction using clinical data and gene expression data compared to prediction using only clinical data. We further conclude that we can obtain improved survival prediction using the RSF model instead of the Cox model. These results are significant because by incorporating more gene expression data with clinical features and using the RSF model, we could develop decision support systems that better utilize heterogeneous information to improve outcome prediction and decision making.

## Introduction

A *clinical decision support system* (*CDSS*) is a computer program, which is designed to assist healthcare professionals and patients with making decisions such as treatment decisions for cancer patients [1]. Studies show that thousands of genes are associated with subtype and prognosis of breast cancer, and particular allele combinations may usefully guide the selection of effective treatment [2]. We have increasingly abundant data on cancer patients that includes clinical features, genomic features, protein abundance, and gene expression. These sources of data provide significant opportunities for developing CDSSs that utilize heterogeneous information sources, such as clinical data and various types of genomic data, to improve outcome prediction and clinical decision making over what is currently possible.

Towards utilizing the available genetic data, efforts have been to predict overall survival, recurrence free survival, and risk of distant metastasis using gene expression data, and a number of commercial products have been developed. An overview of these products appears in [3]. We review four of the more well-known products to set a context for the purpose of the research presented in this paper.

In 2001 Sørlie et al. [4] established a now well-known classification system for breast cancer tumors. Using a sample of 85 tumor samples, from a core set of 8,102 genes, they selected 1,753 genes whose expression varied by at least 4-fold from the median red/green ratio in at least three or more of the samples. Hierarchical clustering was then used to group the gene profiles into 5 major subgroups: Lumina A, Lumina B, ERBB2+, Basal-like, and Normal breast-like. Using 49 of the samples, they developed Kaplan-Meier plots showing overall survival and *relapse-free survival* (*RFS*) for each of these subgroups. These Kaplan-Meir plots showed a significant difference in both types of survival between the subgroups. This result only shows correlation. It does not indicate predictive performance for the subgroups because there was no effort to predict survival for a hold-out group (i.e. a group of samples that was not used to develop the plots).

These subtypes were later extensively studied [5–7]. In 2009 Parker et al. [8] performed studies that led to the commercial product PAM50 [9] Using a sample of 189 tumors, they narrowed down the number of genes needed to predict subclass to 50. Using this sample, They learned Cox proportional hazard [10] RFS models using 1) only ER status, tumor size, and grade; 2) only the subtype; 3) using the subtype and tumor size; and 4) using the subtype, tumor size, and grade. The applied these models to a test data set consisting of 761 tumors. The best results were obtained with subtype, tumor size, and grade (concordance index about equal to 0.67), and the worst results were obtained with only ER status, tumor size, and grade (concordance index about equal to 0.648). Parker et al. [8] conclude that the subgroup is an independent predictor of survival, as also stated in the PAM50 brochure [9]. However, there are many other clinical predictors of survival (See Table 1). To our knowledge, it has not been shown that we obtain improved prediction performance when we use the PAM50 profile in addition to all relevant clinical variables.

**Table 1:** The clinical variables used to predict survival.

Variable	Description	Values
age_at_diagnosis	age at diagnosis of the disease	0–39, 39–54, 54–69, 69–84, 84–100
size	size of tumor in cm	0–20, 20–50, 50–180
lymph_nodes_positive	number of positive lymph nodes	0, 1, 2–3, 4–5, 6–9. ≥10
grade	grade of disease	1, 2, 3
histological	tumor histology	IDC, IDC+ILC, IDC-TUB, IDC-MUC, IDC-MED, MIXED NST AND A SPECIAL TYPE, OTHER, OTHER INVASIVE, INVASIVE TUMOR
ER_IHC_status	ER status	pos, neg
ER_Expr	estrogen receptor expression	+, -
PR_Expr	progesterone receptor expression	+, -
HER2_SNP6_state	HER2 copy number gain or loss	NEUT, GAIN, LOSS
HER2_Expr	HER2 expression	+, -
treatment	Treatment	None, HT, RT, CT, HT/RT, HT/CT, RT/CT, HT/RT/CT
inf_men_status	inferred menopausal status	pre, post
group	characterizes patients by lymph node status, chemo- and hormonal therapy	1, 2, 3, 4, other
stage	composite of size and number of lymph nodes positive	numeric
lymph_nodes_removed	number of lymph nodes removed	numeric
NPI	the Nottingham Prognostic Index, a composite of tumor size, number of lymph nodes positive, and grade	numeric
cellularity	cells seen on histopathology	high, low, moderate
Pam50_subtype	subtype inferred from expression data for 50 genes	Basal, Her2, LumA, LumB, NC, Normal
int_clust_memb	cluster membership according to METABRIC	1, 2, 3, 4, 5, 6, 7, 8, 9, 10
site	collection site information specific to METABRIC	1, 2, 3, 4, 5
Genefu	A composite of other variables used by METABRIC	ER+/HER2-, High Prolif, Low Prolif, ER-/HER2-, HER2+

In 2002 van `t Veer et al. [11] developed a 70 gene expression signature that predicts when the time to distant metastases will be short for lymph node negative tumors. They obtained expression levels for about 25,000 genes from the tumors of 78 sporadic lymph-node negative breast cancer patients. They then used a 3-step supervised classification method to narrow down the number of genes to 70. The signature correctly predicted the outcomes for 65 out of 78 patients. To validate the signature, the classifier was applied to 19 out-of-sample tumors; 17 out of the 19 were correctly classified. This 70 gene classifier evolved into the commercial package MammaPrint [12]. As far as we know, there have been no tests investigating whether equivalent or better prediction could be done using clinical features such as those appearing in Table 1.

In 2004 Paik et al. [13] developed a 21 gene profile that predicts distant recurrence likelihood in patients with breast cancer who have no lymph nodes involved and estrogen-positive tumors. The list of 21 genes and the recurrence-score algorithm were developed by analyzing the results of three independent preliminary studies involving 447 patients and 250 candidate genes. The 21 genes include 16 cancer-related genes and 5 reference genes. They classified patients as low risk if the score was lower than 18, intermediate risk if it was between 18 and 31, and high risk if it was greater than 31. They evaluated the score for tamoxifin-treated patients who were enrolled in the National Surgical Adjuvant Breast and Bowel Project clinical trial B-14. Kaplan-Meier estimates for the patients in the low-risk group who were free of a distant recurrence at 10 years (0.932) were significantly greater than the patients in the high-risk category (0.695). This 21 gene profiling scheme led to the commercial package Oncotype DX [14], which is intended to be used by women with early-stage, node-negative, ER+ invasive breast cancer. It not only predicts recurrence but also estimates the likelihood of chemotherapy benefit. To our knowledge, there have been no studies conducting whether this test yields improved prediction performance over just using clinical variables like those listed in Table 1.

In 2011 Filibits et al. [15] predicted the likelihood of distant recurrence in ER+, HER2 negative breast cancer patients with adjuvant endocrine therapy. The system, which was developed by analyzing 964 breast cancer patients, provides the Endopredict (EP) score obtained from 8 cancer-related genes and 3 normalization genes, tumor size, and the number of lymph nodes involved. The score was validated using patients analyzed in the ABCSG translational research program (abcsg.research). The validation groups were ABCSG-6 which contains 378 patients and ABCSG-8 which contains 1,324 patients. Finally they used Cox regression to develop a prediction model containing only the variables age, quantitative ER (HIC), and Ki67. Furthermore they evaluated the Adjuvant!Online [16] score, which is obtained from clinical variables. In tests using the same validation groups, they obtained better concordance indices using the EP score than the other methods alone, and combinations of the other methods. Endopredict is now a commercial package [17].

The EP score is different from the other methods discussed because it includes two clinical features. Furthermore, it was shown to outperform clinical prediction using Adjuvant!Online. However, it was not evaluated relative to a comprehensive list of clinical features like those shown in Table 1.

The four methods just discussed, and other such methods, suffer from one or more of the following difficulties: 1) They were developed and tested with fairly small data sets; 2) They only apply to specialized subsets of patients; 3) As discussed in [11] these signatures do not always include genes widely believed to be involved in breast cancer. 4) They perform prediction using the Cox proportional hazard model. However, the purpose of that model is more to identify covariates than to predict survival. The *random survival forest method* (*RSF*) [18] has been shown to significantly outperform the Cox model in several studies [19,20]. 5) Most importantly, there was not sufficient testing to see if a complete set of clinical predictors could make the same predictions as the gene expression signatures.

The studies presented here address the 4^th^ and 5^th^ issues. That is, we consider it still an open question as to whether we can perform better outcome prediction utilizing high-dimensional gene expression data in addition to clinical data. Specifically, we investigate whether a system that uses both a complete set of clinical features and gene expression profiles can improve survival prediction relative to one that uses only a complete set of clinical features. Furthermore, we investigate whether the random survival forest method outperforms the Cox model at cancer survival prediction.

The *Molecular Taxonomy of Breast Cancer International Consortium* (*METABRIC*) data set [21] has data on 1981 breast cancer tumors. Features include 21 clinical features, expression levels for 16,384 genes, and overall survival. The objective of this investigation is to compare the survival prediction performance of the Cox proportional hazards model and the RSF model using the clinical data and the gene expression data in the METABRIC data set to their performance using only the clinical data. *The central hypotheses are 1) We can obtain better survivorship prediction performance using both clinical features and gene expression data relative to using only clinical features; and 2) We can obtain better survivorship prediction using the RSF model relative to using the Cox model*.

Central to a CDSS for breast cancer patients is a component that predicts outcomes such as overall survival, distant metastasis survival, local breast recurrence, and local lymph node recurrence. Our ideal system would include all relative clinical variables and all relevant genetic variables, and be applicable to all types of breast cancer. Our results are significant to the medical community because they help to answer the question as to whether gene expression data would be useful to such a system, and whether it would be preferable to use the RSF model instead of the Cox Model.

Since we evaluate the RSF and Cox model, we close this section by briefly reviewing them. A survival prediction model learns a *survival function* from survival data. Such a function predicts the probability of surviving past each point in time based on an individual’s covariates (predictors). For a given individual, the resultant function of time is called a *survival curve*. The hazard function λ(*t*) is the probability of dying at time *t* given that one has not died before time *t*. The hazard ratio is the ratio of λ(*t*) to a reference hazard function λ_0_(*t*). The Cox proportional hazards model is a linear model for the log of the hazard ratio in terms of the covariates. When we learn the survival function, we learn the linear coefficients of the covariates. The RSF model works quite differently. A *survival tree* is a type of classification tree. Each node in a classification tree makes a choice based on the value of a covariate until the leaf classifies the instance. The tree is developed by starting at the root and determining the covariate that best identifies the instance according to some criterion such as information gain. Recursively, the tree is then built to the roots. In the RSF model, *M* bootstrap samples are obtained from the original data. Each bootstrap sample excludes on the average 37% of the data. A survival tree is grown for each bootstrap sample. At each node of the tree, *N* candidate covariates are randomly selected. The node is split using the covariate that maximizes survival difference between daughter nodes. The tree is grown until a leaf node has no less than *D* > 0 unique deaths. Finally, the *cumulative hazard function* (*CHF*) is calculated for the tree. The average CHF is taken over all bootstrap samples, and the prediction error is calculated using the data that was left out.

## Methods

We evaluated survival prediction performance of the Cox proportional hazards model [10] and the RSF model [18] using the METABRIC data set [21] which concerns primary breast tumors. That data set has data on 1981 tumors. Features include 21 clinical features and expression levels for 16,384 genes. The *Multiple Imputation with Diagnostics* (MI) Package [22,23] was used to impute missing values in the data set.

All gene expression levels were discretized to values *low*, *medium*, and *high* using the equal width discretization technique, which discretizes the data into partitions of *M* equally sized intervals (*M* = 3 in our application).

Table 1 shows the clinical variables and their values used in our analysis. We transformed the data in three of these variables from their original METABRIC values using a combination of domain knowledge and the equal distribution discretization strategy. The transformations are as follows:

*age_at_diagnosis*: We discretized this variable to the ranges shown based on a combination of the equal distribution discretization technique and breast cancer expert knowledge.
*size*: We discretized this variable to the three standard ranges shown.
*lymph_nodes_positive*: We grouped this variable into the six ranges shown.

Furthermore, the data set has the following two fields:

*day*: This field is a number of days.
*status*: This field's value is *dead* if the patient died *day* days after the initial consultation, and its value is *alive* if the patient was last seen *day* days after initial consultation (and therefore was known to be alive at that time).

Any patient, whose *status* field contains the value *alive*, is right censored. Right censored means the patient left the study. All we know is that the patient was still alive the last time the patient was seen. We created a table as shown in Table 2. Patient 2 was found to be *dead* in *Year*_2_. So *Year*_2_ and all subsequent years in Table 2 have value *dead*. Patient 3 was last seen in year 2, and was alive. So we don't know the status of Patient 3 in subsequent years, and that patient is right censored.

**Table 2:** A table developed from the METABRIC data set.

Patient	*X*_1_	*X*_2_	…	X_n_	*Year*_1_	*Year*_2_	*Year*_3_	…	*Year*_14_	*Year*_15_
1					alive	alive	alive		alive	alive
2					alive	dead	dead		dead	dead
3					alive	alive	NA		NA	NA
…										

The METABRIC data set contains the variable Pam50_subtype, which is the subtype inferred from expression data for 50 genes as discussed above [8,9]. Since this variable is a composite of gene expression data, we removed it to obtain data that was only clinical. We call that data set *Clinical_Only*. We obtained data including gene expression data in two ways. We used all the clinical data in the METABRIC data set including Pam50_subtype. We call that data set *Clinical_PAM*. We used the clinical data without Pam50_subtype, but with the expression levels of all 16,384 genes. We call that data set *Clinical_Gene*. We evaluated the Cox model and the RSF model when they utilized these three data sets.

There are way too many genes to try to perform prediction using all of them directly when utilizing *Clinical_Gene*. We limited the number of features by pre-processing the data set using ReliefF. The ReliefF algorithm [24] ranks a set of possible predictor variables in terms of how well they predict the target variable. This algorithm does not look at each predictor individually. Rather, it is aware of contextual information, and estimates predictive strength in light of a predictor’s interactions with other predictors. The basic algorithm, called Relief assumes the target variable is binary, and that there are no missing data. This is the situation in our studies. These restrictions are removed in ReliefF, and you are referred to [24] for this more general algorithm. We present the Relief algorithm that we used next.

**Algorithm:** Relief

**Input:** A set of data items. Each data item contains values of predictor variables and a value of a binary target variable *T*.

**Output:** For each predictor *F*, a weight *W*[*F*] that estimates the predictive strength of *F* for *T*.

**for** each predictor *F*

*W* [*F*] = 0;

**endfor**

**repeat** *m* times

   randomly select a data item *D*;

   determine nearest hit *H* to *D*;

   determine nearest miss *M* to *D*;

**for** each predictor *F*

*W* [*F*] = *W* [*F*] – *Diff* (*F,D,H*)/ *m* + *Diff* (*F,D,M*)/ *m*;

**endofor**

**endrepeat**

The function *Diff* in Algorithm Relief is as follows;

$$Diff\left(F,D,X\right)=\{\frac{\begin{array}{r} \text{0 if }D\text{ and }X\text{ have the same value for }F\text{;} \end{array}}{1\text{ otherwise}.}$$

By “nearest hit *H* to *D*”, we mean the data item *H* closest to *D* that has the same value of the target as *D*; and by “nearest miss *M* to *D*”, we mean the data item *M* closest to *D* that has the has a different value of the target than *D*. If *D* and *H* have different values for predictor *F*, then *F* separates two close data items with the same value of the target, which indicates *F* is not a good predictor; so we decrease the weight. On the other hand, if *D* and *M* have different values for predictor *F*, then *F* separates two close data items with different values of the target, which indicates *F* is a good predictor; so we increase the weight.

When ReliefF is employed, different sized sets of top predictive features can be used to perform prediction, and the set of features that yields the best performance is the one that is used in the prediction system. We used sets of 30, 50, 100, and 150 top predictive features. We did this in two ways: *Method 1* combined the clinical features and the gene expression data, and then used ReliefF to extract features. *Method 2* extracted gene expression features from the gene expression data, and then combined those features with all 21 clinical features. When using this latter method, we only extracted 9, 29, 79, and 129 features so that the total number of features was still 30, 50, 100, and 150.

We developed 5 year, 10 year, and 15 year survival functions using each of the methods. The 5 year survivor functions were learned only from survival information obtained from the first 5 years of post-initial visit information, the 10 year survivor functions were learned only from the first 10 years of information, and the 15 year survivor functions were learned only from the first 15 years of information. When using ReliefF, we used as our target survivorship at 5 years to extract features for the 5 year survival functions, at 10 years to extract features for the 10 year survival functions, and at 15 years to extract features the 15 year survival functions. Note that we did not utilize any assumed biological knowledge concerning which genes might be good predictors. We relied only on machine learning to find good predictors. In this way our results do not depend on any previous analyses.

In *k-fold cross validation*, we divide the data into *k* partitions of the same size. For each partition *j* we learn a model using the data in the remaining *k*-1 partitions, and we then apply the model to partition *j*. For each survival timeframe (5 year, 10 year, or 15 year) we compared methods using 5-fold cross validation. Specifically, for each method, survival timeframe, and partition, we learned a survival function from the individuals in the remaining 4 partitions. We then used that function to develop a survival curve for each individual in the given partition. Next, we computed the concordance for the individuals. The concordance index is the probability that, given two randomly drawn individuals, the individual who has the event first has a worse survival curve. By “worse” we mean the area under the curve is smaller. To estimate the concordance index, we pair each individual *j* known to be dead, with each individual *k* known to be alive later than *j* or known to die later than *j*. Let *area*_*j*_ be the area under the survival curve for individual *j*, and *area*_*k*_ be the area under the survival curve for individual *k*. If *area*_*j*_ < *area*_*k*_, we add 1 to a variable *Total*. Finally, we divide *Total* by the number of pairs to obtain the concordance index. We statistically compared the concordance indexes for two methods using the chi-square test.

## Results

S1–S4 Tables show the raw results. That is, they show results for both Method 1 and Method 2, and for all values of the number of features obtained from ReleifF.

**Fig 1:**
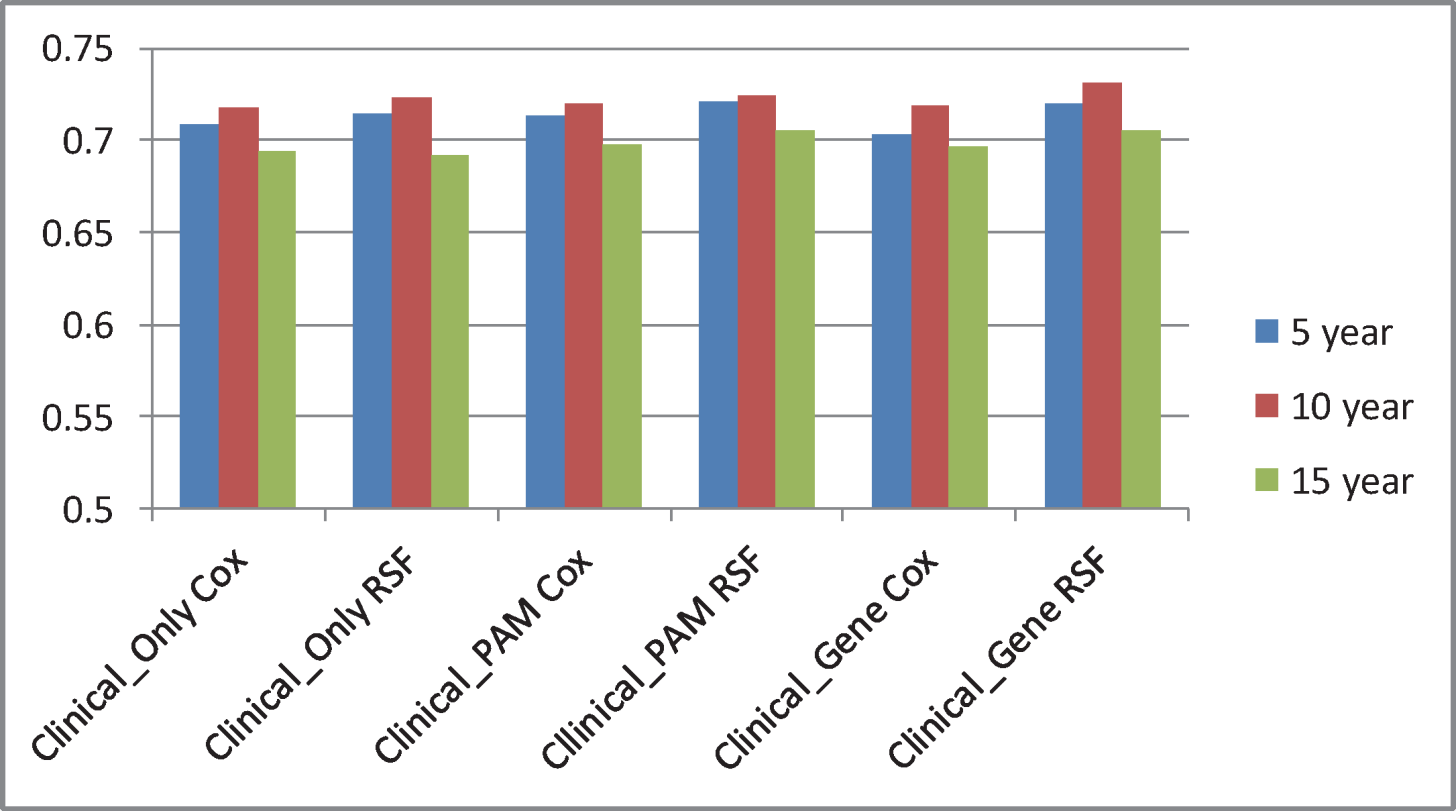
Comparison of the Cox concordance index results and the RSF concordance index results for each type of data.

Table 3 and Fig. 1 compare the performances of the Cox model and the RSF model, while showing only the best results for *Clinical_Gene*. For both the Cox model and the RSF model these best results were obtained using Method 2. We see that the RSF model performs significantly better (p < 2.2 × 10^–16^) than the Cox model in every case except for *Clinical_Only* and 15 years, and this latter result is not significant. The most improvement for the RSF model was realized with *Clinical_Gene*. The Cox model is based on linear regression and does not do shrinkage to handle a large number of variables such as that done by a regularized technique such as Lasso [25]. This would explain why it performs poorly when given many features.

**Table 3:** Comparison of the Cox concordance index results and RSF concordance index results for each type of data.

Year	Clinical_Only		Clinical_PAM		Clinical_Gene	
	Cox	RSF	Cox	RSF	Cox	RSF
5	0.709	0.714	0.713	0.721	0.703 (30)	0.720 (150)
10	0.718	0.723	0.720	0.724	0.719 (30)	0.731 (50)
15	0.694	.0692	0.698	0.705	0.696 (30)	0.706 (100)

For *Clinical_Gene* these are the best results obtained by the model. They were all obtained with Method 2. The number in parenthesis shows the number of features obtained from ReliefF that yielded the best results. Other than the *Year* 15 entry for *Clinical_Only*, the comparison of the Cox results and RSF results is significant at p < 2.2 × 10^–16^.

**Fig 2:**
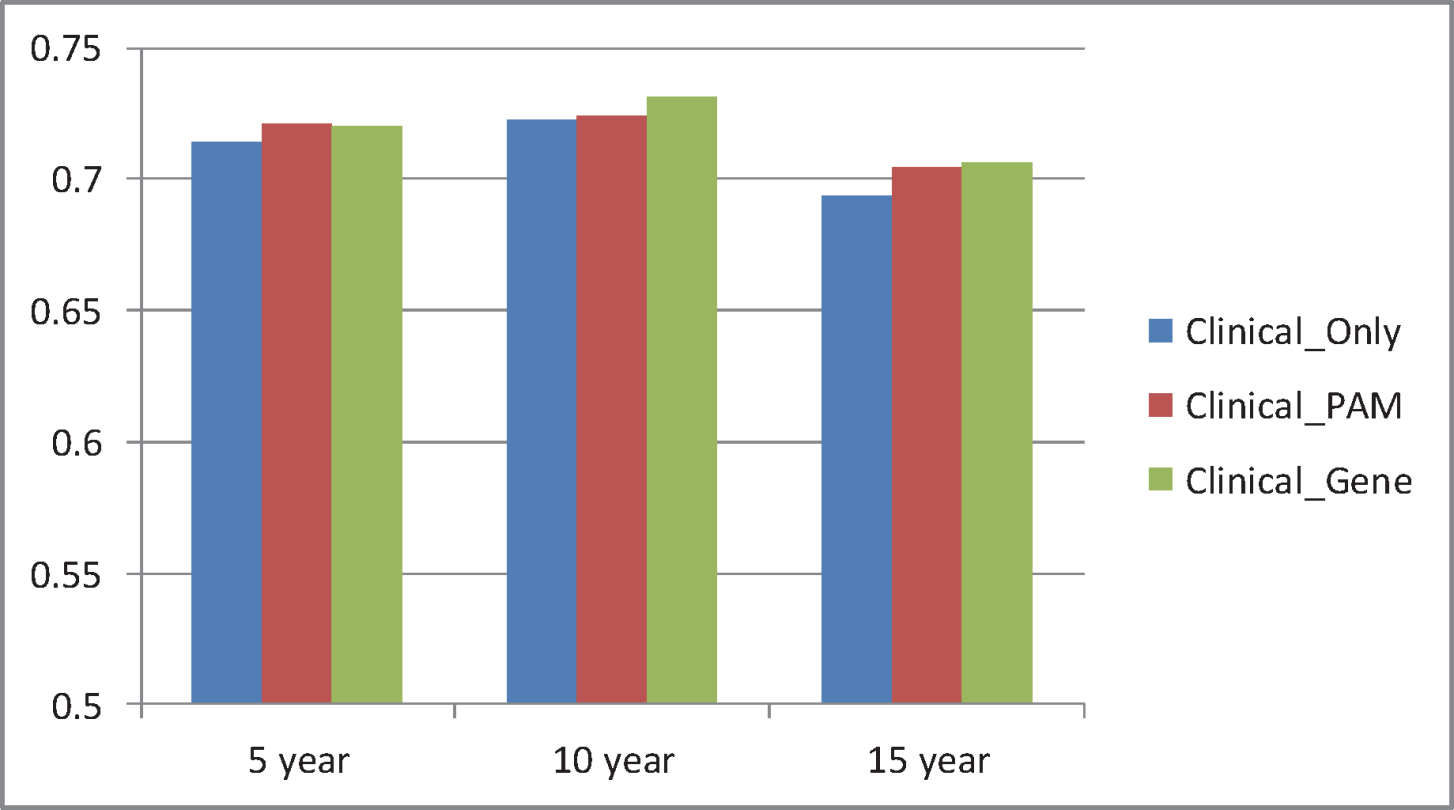
Comparison of the best results obtained for each data set over both models (Cox and RSF) and all values of the number of features provided to ReliefF.

We are most interested in comparing the best results obtained for each data set over both models (Cox and RSF) and all values of the number of features provided to ReliefF because we would use the best results in an actual prediction method. Table 4 and Fig. 2 show those results. *Clinical_Gene* performs significantly better than *Clinical_Only* for all values of *Year* (p < 2.2 × 10^–16^). *Clinical_Pam* performs about as well as *Clinical_Gene* for 5 years and 15 years, but barely performs better than *Clinical_Only* for 10 years.

**Table 4:** Comparison of the best concordance index results obtained for each data set over both models (Cox and RSF) and all values of the number of features provided to ReliefF.

Year	Clinical_Only	Clinical_PAM	Clinical_Gene	p-value PAM	p-value Gene
5	0.714	0.721	0.720	1.17×10^–14^	6.77×10^–14^
10	0.723	0.724	0.731	0.251	< 2.2 × 10^–16^
15	0.694	0.705	0.706	p < 2.2 × 10^–16^	< 2.2 × 10^–16^

The 5th column shows the p-values obtained when *Clinical_Only* is compared to *Clinical_PAM*. The 6th column shows the p-value when *Clinical_Only* is compared to *Clinical_Gene*.

**Fig 3:**
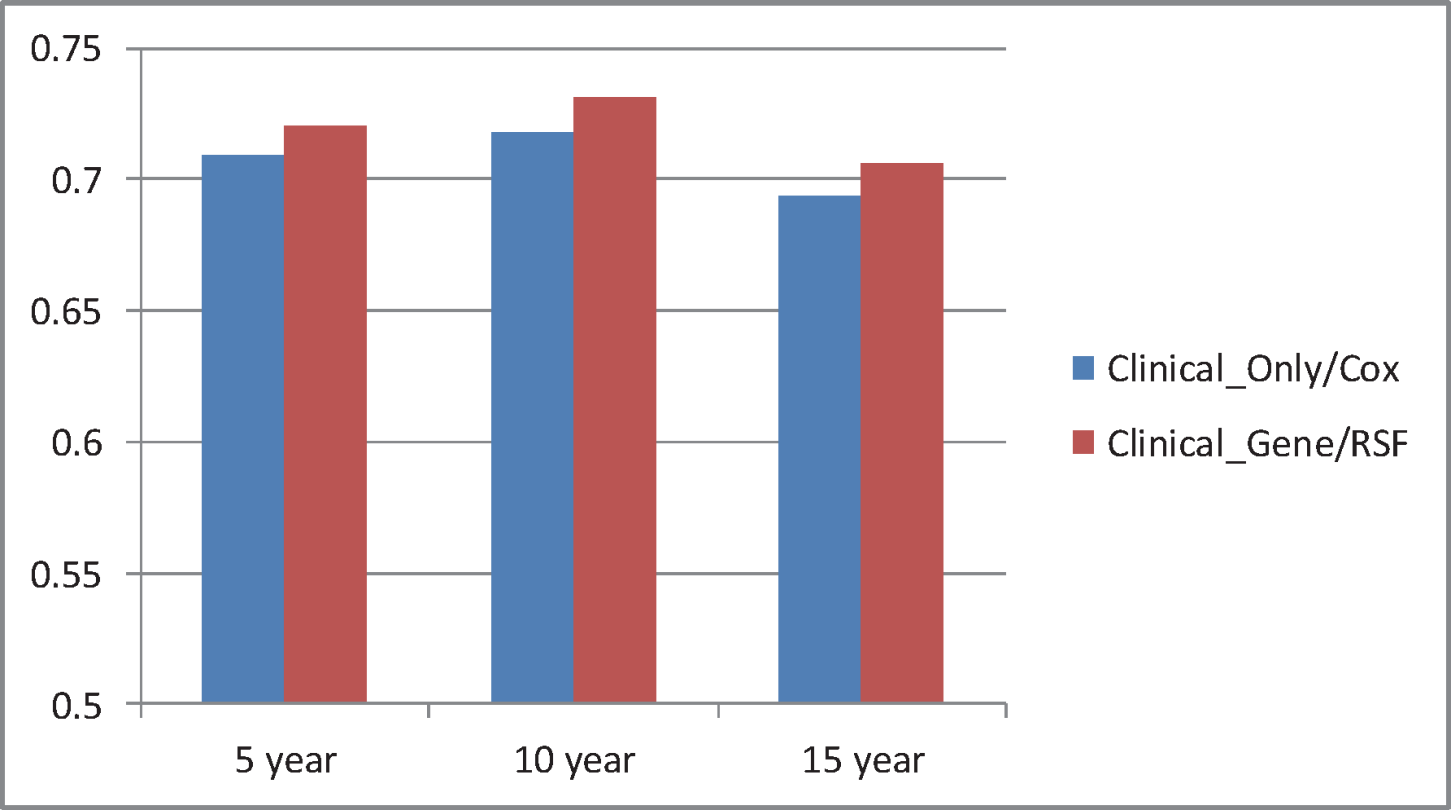
Comparison of the best concordance index results obtained using *Clinical_Gene* and the RSF model to the concordance index results obtained using *Clinical_Only* and the Cox model.

Our chief purpose was to investigate whether we would obtain better performance by including gene expression data and by using the RSF model instead of using only clinical data and using the Cox model. Table 5 and Fig. 3 compare the two. We see that Clinical_Gene using the RSF models is up to almost 2% more accurate than Clinical_Only using the Cox model.

**Table 5:** Comparison of the best concordance index results obtained using *Clinical_Gene* and the RSF model to the concordance index results obtained using *Clinical_Only* and the Cox model.

Year	Clinical_Only/Cox	Clinical_Gene/RSF	Percent Increase
5	0.709	0.720	0.015
10	0.718	0.731	0.018
15	0.694	0.706	0.017

All results are significant at p < 2.2 × 10^–16^.

**Fig 4:**
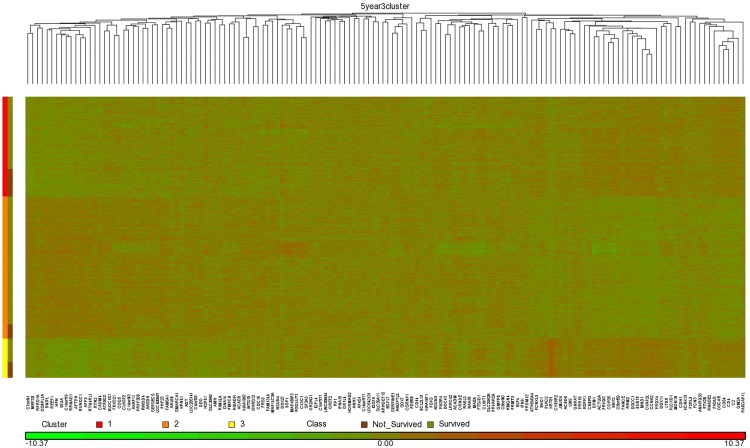
Heat map clustering 1981 breast cancer tumors and the top 150 genes learned using ReliefF from the entire data set for 5 year survival prediction.

**Fig 5:**
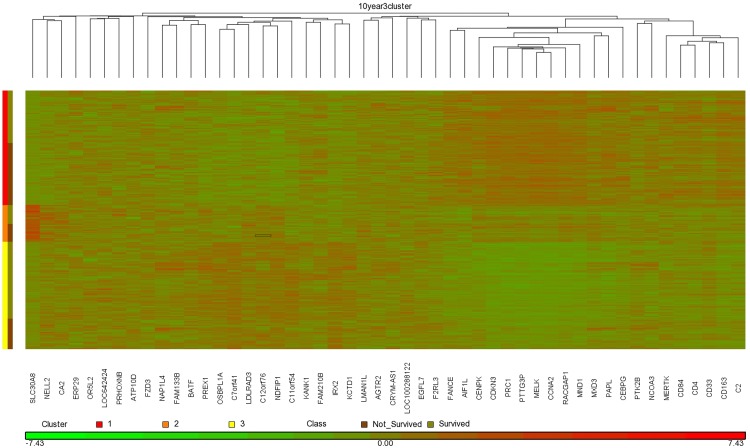
Heat map clustering 1981 breast cancer tumors and the top 50 genes learned using ReliefF from the entire data set for 10 year survival prediction.

**Fig 6:**
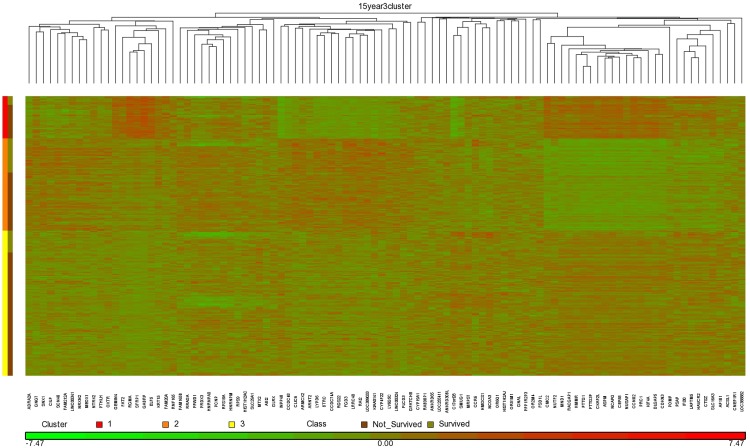
Heat map clustering 1981 breast cancer tumors and the top 100 genes learned using ReliefF from the entire data set for 15 year survival prediction.

Our studies were done using 5-fold cross validation. In general, if we were to then use the method in an actual production system, we would learn from the entire data set. S5 Table shows the top 150 features extracted by ReliefF from the entire data set for Method 1 and Method 2, and for 5 year, 10 year, and 15 year prediction. We obtained our best overall results using the RSF model and Method 2, and using 150 genes for 5 year prediction, 50 genes for 10 year prediction, and 100 genes for 15 year prediction. Figs. 4, 5, and 6 show heat maps concerning these top genes when ReliefF learns from the entire data set. These heat maps were created using the software package Partek (http://www.partek.com/), which allows one to perform either hierarchical clustering or partition clustering. Hierarchical clustering break up the data in to a hierarchy of clusters. Partitional clustering divided the data into mutually disjoint partitions. We did partition clustering for the individuals on the y-axis and hierarchical clustering for the genes on the x-axis. In Figs. 4–6 we also show the individuals in each cluster who survived and did not survive. These results are consistent with Table 4, which shows that *Clinical_Gene* exhibits the least improvement relative to *Clinical_Only* in the case of 5 year survival prediction. Looking at the 5 year survival heat map in Fig. 4, we see that the fraction of individuals not surviving is the not much different in the three clusters. However, the 10 year survival heat map in Fig. 5 shows that individuals in Cluster 1 are much less likely to survive than individuals in the other two clusters. Similarly, the 15 year survival heat map in Fig. 5 shows that individuals in Clusters 1 and 3 are less likely to survive than individuals in Cluster 2.

It is interesting to investigate which genes were found to be predictive for two or more time frames. Table 6 shows the genes, from the top 150 genes extracted by ReliefF, which the time frame pairs have in common. Time frames 5 year and 10 year share 18 predictive genes, time frames 10 year and 15 year share 12 predictive genes, and time frames 5 year and 15 year share only 2 predictive genes. All three time frames share only the predictive genes MND1 and CKAP2L. The *MND1* gene has been shown to be essential for meiotic recombination between homologous chromosomes [26]. Its disruption results in severe defects in homologous chromosome synapsis and an early-stage failure in meiotic recombination. CKAP2L has recently been shown to be an independent prognostic marker for RFS in early-stage breast cancer [27]. The fact that the 5 year and 10 year timeframes share so few genes is interesting in its own right, and indicates that many genes related to short term survival might be different from those related to long term survival.

**Table 6:** The predictive genes, from the top 150 genes extracted by ReliefF, that the time frame pairs have in common.

5 / 10 year	5 / 15 year	10 / 15 year
OR2AG1	MND1	MND1
EGFL7	CKAP2L	CDKN3
SLC30A8		PTTG3P
RRM2		PRC1
MND1		RACGAP1
HSPB1		NCOA3
C2		HMGCS1
FANCE		CKAP2L
OSBPL1A		CDCA5
CEBPG		CEP55
F2RL3		KIF4A
CCNA2		SFRP1
CKAP2L		
PTK2B		
C7orf41		
CD84		
CD4		
DSCC1		

It is also revealing to investigate the clinical features chosen by Method 1. Table 7 shows them. In the case of both 5 year and 10 year survival, *lymph_nodes_positive* and *group* are the top two features. However, in 15 year survival, *lymph_nodes_positive* does not appear at all, and there is only one clinical feature in the top 50 features. That feature is the *Nottingham prognostic index* (*NPI*), which is calculated using the size of the lesion, the number of lymph nodes involved, and the grade of the tumor; and which therefore includes the information in the feature *lymph_nodes_positive*. A similar situation holds for the 15 year survival training sets (not shown). Namely, NPI is the top clinical feature in all of them, and its average location is 25^th^. Only one of them has another clinical feature (“group” which appears 11^th^) in the top 50 features, and one has no clinical features in the top 50 features. Yet, as we can see from S2 Table, we can get fairly good prediction (concordance index equal to 0.654) using the top 50 features. This result indicates we may be able to predict much of long term survival using gene expression signatures.

**Table 7:** The clinical features extracted by ReliefF in the case of Method 1.

5 Year		10 Year		15 Year	
Rank	Feature	Rank	Feature	Rank	Feature
1	lymph_nodes_positive	1	group	5	NPI
2	group	2	lymph_nodes_positive	77	group
49	stage	3	NPI	129	treatment
110	size	12	size	138	int_clust_memb
		14	site		
		102	stage		
		145	age_at_diagnosis		

The *Rank* is where the feature occurs in the top 150 features.

## Discussion

We compared survival prediction using both clinical data and gene expression data to survival prediction using only clinical data. We obtained significantly better results when we used both clinical data and gene expression data relative to when we used only clinical data. When we included Pam50_subtype, which is a composite of the expression data for 50 genes, with the clinical features, our results were significantly better only for 5 year and 15 year prediction. When we used ReliefF to choose good gene expression predictors, the results were significant for 5 years, 10 year, and 15 year prediction. This information is meaningful because often 10 year survival is the most utilized survival criterion.

We also compared survival prediction using both the Cox model and the RSF model, and we obtained significantly better performance using the RSF model. This result is also notable because the Cox model is often used to do survival prediction in current systems (as noted in the Introduction Section). Furthermore, our results indicate that the Cox model cannot handle a large number of covariates, and might explain why existing methods (as discussed in the Background Section) limit the number of genes or group them into subtypes. As noted in the Introduction Section, studies show that thousands of genes are associated with subtype and prognosis of breast cancer [2]. As also noted, current signatures do not always include genes widely believed to be involved in breast cancer [11]. With the power of the RSF model, we can possibly include all relevant features and improve prediction.

Our results indicate that we can improve prediction performance by using gene expression date beyond the subtype composites in PAM50. Future research can investigate feature selection further. First, we can investigate whether we get similar, or even improved, results using strategies other than ReliefF to locate good predictors. For example, we could use the Bayesian network-based methods that look for interacting causes of a target [28]. We can investigate how gene expression data and clinical features might interact. We can see if the RSF method remains better than the Cox method when these other strategies are used. Second, we can investigate whether we can improve prediction further by including *copy number variations* (*CNV*) and *copy number alterations* (*CNA*). We can utilize both machine learning knowledge and biological knowledge in our choice of genetic features. Finally, since we have learned that the prediction method which is used can make a substantial difference, we can investigate using other survival prediction methods such as the recently developed Bayesian network based method S_EBMC [28].

We obtained an interesting result when we applied ReliefF in Method 1 to simultaneously learn good clinical and gene expression predictors. That is, our results show that very few clinical features are needed for 15 year prediction. This would indicate that much of long-term survival can be explained by gene expression signatures. If someone survives long-term, it is likely they died of something other than breast cancer (although our data is not able to support this claim). So, perhaps long-term survival in general can be predicted by gene expression signatures, which opens the possibility of increasing longevity by targeting gene expression.

## Supporting Information

S1 Table Concordance index results for the Cox model Using Method 1. (DOC)

S2 Table Concordance index results for the RSF model Using Method 1. (DOC)

S3 Table Concordance index results for the Cox model Using Method 2. (DOC)

S4 Table Concordance index results for the RSF model Using Method 2. (DOC)

S5 Table Lists of the 150 features extracted by ReliefF for Methods 1 and 2 and for 5 year, 10 year, and 15 year prediction. (XLSX)
