# Supplementary material for: Study of Integrated Heterogeneous Data Reveals Prognostic Power of Gene Expression for Breast Cancer Survival
Source: PLoS One. 2015 Feb 27;10(2):e0117658. doi: 10.1371/journal.pone.0117658 (PMC4344205; doi:10.1371/journal.pone.0117658)
Supplement: S2 Table — (DOC) [file pone.0117658.s002.doc]

**Table S2** Concordance index results for the RSF model Using Method 1. The 1^st^ column shows the number of features extracted by ReliefF. The 3^rd^ column shows results obtained with all the clinical features excluding Pam50_subtype; the 4^th^ column shows results obtained with all the clinical features including Pam50_subtype; the 5^th^ column shows results obtained with Method 1 excluding Pam50_subtype. The * denotes where the best results for the 5^th^ column were obtained.

| # Features | Year | Clinical_Only | Clinical_PAM | Clinical_Gene |
| --- | --- | --- | --- | --- |
| 30 | 5 | 0.714 | 0.721 | 0.692 |
| 30 | 10 | 0.723 | 0.724 | 0.720 |
| 30 | 15 | 0.692 | 0.705 | 0.645 |
| 50 | 5 | 0.714 | 0.721 | 0.695 |
| 50 | 10 | 0.723 | 0.724 | 0.722 |
| 50 | 15 | 0.692 | 0.705 | 0.654 |
| 100 | 5 | 0.714 | 0.721 | 0.703 |
| 100 | 10 | 0.723 | 0.724 | 0.722 |
| 100 | 15 | 0.692 | 0.705 | 0.663 |
| 150 | 5 | 0.714 | 0.721 | 0.708 * |
| 150 | 10 | 0.723 | 0.724 | 0.727 * |
| 150 | 15 | 0.692 | 0.705 | 0.669 * |
